# Transcriptomic analysis of atopic dermatitis in African Americans is characterized by Th2/Th17-centered cutaneous immune activation

**DOI:** 10.1038/s41598-021-90105-w

**Published:** 2021-05-27

**Authors:** Shannon Wongvibulsin, Nishadh Sutaria, Suraj Kannan, Martin Prince Alphonse, Micah Belzberg, Kyle A. Williams, Isabelle D. Brown, Justin Choi, Youkyung Sophie Roh, Thomas Pritchard, Raveena Khanna, Amarachi C. Eseonu, Jaroslaw Jedrych, Carly Dillen, Madan M. Kwatra, Anna L. Chien, Nathan Archer, Luis A. Garza, Xinzhong Dong, Sewon Kang, Shawn G. Kwatra

**Affiliations:** 1 Department of Dermatology, Johns Hopkins University School of Medicine, Baltimore, MD USA; 2 Department of Biomedical Engineering, Johns Hopkins University School of Medicine, Baltimore, MD USA; 3 Department of Anesthesiology, Duke University School of Medicine, Durham, NC USA; 4 The Solomon H. Snyder Department of Neuroscience, Center for Sensory Biology, Johns Hopkins University School of Medicine, Baltimore, MD USA

**Keywords:** Computational biology and bioinformatics, Immunology, Medical research

## Abstract

Atopic dermatitis (AD) often presents more severely in African Americans (AAs) and with greater involvement of extensor areas. To investigate immune signatures of AD in AAs with moderate to severe pruritus, lesional and non-lesional punch biopsies were taken from AA patients along with age-, race-, and sex-matched controls. Histology of lesional skin showed psoriasiform dermatitis and spongiotic dermatitis, suggesting both Th2 and Th17 activity. Gene Set Variation Analysis showed upregulation of Th2 and Th17 pathways in both lesional versus non-lesional and lesional versus control (p < 0.01), while Th1 and Th22 upregulation were observed in lesional versus control (p < 0.05). Evidence for a broad immune signature also was supported by upregulated Th1 and Th22 pathways, and clinically may represent greater severity of AD in AA. Furthermore, population-level analysis of data from TriNetX, a global federated health research network, revealed that AA AD patients had higher values for CRP, ferritin, and blood eosinophils compared to age-, sex-, and race-matched controls as well as white AD patients, suggesting broad systemic inflammation. Therefore, AA AD patients may feature broader immune activation than previously thought and may derive benefit from systemic immunomodulating therapies that modulate key drivers of multiple immune pathways.

## Introduction

Atopic dermatitis (AD) is a complex, inflammatory skin disease that presents with a wide range of endotypes underlying differences in disease presentation^1–4^. AD is generally thought to follow a classic pattern of distribution, with lesions symmetrically affecting flexural surfaces^5^. However, there are clinically meaningful racial differences in disease prevalence and clinical presentation^6–12^. Relative to European American patients, African American (AA) patients typically present with increased papular lesions, as well as greater involvement of the extensor surfaces^5,9,10,13^. AA patients are also more likely to present with a treatment-resistant lichenified phenotype, with noted keratinocyte proliferation, hyperkeratosis, and epidermal hyperplasia^2^.

With the heterogeneity present in the clinical presentation of AD, additional studies are needed to better understand the molecular differences responsible for these unique clinical endotypes, especially in AA patients who are disproportionately affected by AD but are generally understudied. Although AD is thought to be primarily Th2-driven, recent studies have suggested that there are disease subtypes with distinct cytokine signatures. These studies have largely been conducted in European American adults and large gaps remain in the understanding of AD in AA patients at the molecular level. Additionally, AA patients with AD often face unique treatment challenges and do not respond to treatments in the same manner as European American patients. Studies focusing on AA patients with AD are urgently needed to provide insights into AA AD at the molecular level and elucidate potential targeted therapeutic strategies^2,4,14,15^. Thus, we investigated the immune signature of AD in AAs with moderate to severe pruritus. We hypothesized that atopic dermatitis in AA individuals would demonstrate broad cutaneous immune activation, including the Th2 and Th17 axes, given their more hyperkeratotic and lichenified phenotype.

## Methods

### Patient characteristics

This research was a cross-sectional study conducted at Johns Hopkins University (Baltimore, MD, USA). A total of 18 skin biopsies were collected from AA AD patients, and age-, sex-, and race-matched controls with clinically determined healthy skin, and no known cutaneous disease were included in the study. Patients with AD and moderate to severe pruritus were diagnosed using the Hanifin and Rajka criteria^16^. None of the patients were receiving systemic immunosuppressive therapy or applying topical immunomodulating agents to the biopsy site. Patients were recruited from the dermatology outpatient clinics at Johns Hopkins Hospital (August 2018–April 2019). Adult patients diagnosed with AD with self-reported moderate or severe itch corresponding to a score of 2 or 3 on the Verbal Rating Scale (VRS)^17^ were included in the study. Healthy controls were recruited to match the AD participants by age (± 9 years), sex, and race.

Patient biopsy sites were determined via clinical presentation and patient-reported itch. Pruritic lesional and non-pruritic, non-lesional 4-mm skin punch biopsy specimens were collected from each patient with AD. Biopsy locations of healthy patients corresponded to biopsy sites of their matched AD patient. Each biopsy was bisected. One-half biopsy was snap-frozen in liquid nitrogen and stored at − 80 °C. One-half biopsy was placed in 10% neutral buffered formalin at room temperature.

### Ethic declarations

This study was approved by the Johns Hopkins School of Medicine Institutional Review Board (IRB), and all participants signed written informed consent forms. The study was performed in accordance with the institution’s guidelines and regulations. Use of TriNetX was exempt from IRB approval due to use of de-identified aggregate data.

### Histology

Biopsies fixed in 10% neutral buffered formalin fixative were embedded in paraffin and cut into slides. Slides were stained with hematoxylin and eosin (H&E) and assessed for inflammation by a blinded dermatopathologist. The assessment included epidermal thickness, general inflammation, specific inflammatory cell presence and localization, and additional findings. Epidermal thickness was measured at the thickest suprapapillary epidermal plate. General inflammation was graded on a scale from 0 to 3 (0 = no infiltrate, 1 = mild infiltrate, 2 = moderate infiltrate, 3 = prominent infiltrate). Specific inflammatory cells found on histology included lymphocytes, neutrophils, eosinophils, mast cells, and plasma cells.

### RNA-sequencing

Messenger RNA-seq (mRNA) compared gene expression in pruritic, lesional AD skin and non-pruritic, non-lesional AD skin to controls matched for age, race, sex, and biopsy site. Skin specimens obtained via punch biopsy performed on each participant were snap-frozen in liquid nitrogen and stored at − 80 °C. Total RNA was extracted and assessed for quality on Fragment Analyzer (Agilent Technologies) and concentration on Qubit 2.0 (ThermoFisher Scientific). The mean RNA yield was 94.54 ng/μl and mean RNA quality number was 4.24. Under a Material Transfer Agreement, samples were transferred to the SGT Sequencing and Genomic Technologies Laboratory at Duke University, where RNA-seq libraries were prepared with KAPA Stranded mRNA-Seq Kit (Roche). Sequencing was performed on an Illumina NovaSeq 6000 sequencer. Input RNA amount per sample for RNA-seq was 200 ng of total RNA.

### Systemic inflammatory markers

We queried TriNetX, a global federated health research network providing access to electronic medical records from approximately 71 million patients in 49 large Healthcare Organizations. Adult patients with AD were identified using the International Classification of Disease, Tenth Revision (ICD-10) code L20. Only patients with two or more ICD-10 codes for AD were included to increase diagnostic accuracy. Control patients included those with no ICD-10 codes for AD. Laboratory values were restricted to ± 3 months the initial AD diagnosis and included erythrocyte sedimentation rate (ESR), C-reactive protein (CRP), ferritin, and blood eosinophil %.

### Statistical analysis

The resulting data were processed using the fastp toolkit to trim low-quality bases and Illumina sequencing adapters from the 3′ end of the reads^18^. Only reads that were 20 nucleotides or longer after trimming were kept for further analysis. Using the STAR RNA-seq alignment tool, the acceptable reads were mapped to the GRCh38v93 version of the human genome and transcriptome^19,20^. If reads mapped to a single genomic location, they were kept for subsequent analysis. The average unique mapping rate per sample was 80.84%. Gene counts were compiled using the feature Counts tool^21^. Genes that had at least 10 reads in any given library were then used in subsequent analysis. The DESeq2 Bioconductor package with the R statistical programming environment was used to achieve normalization and differential expression of the RNA-seq data^22,23^. The false discovery rate (FRD) was calculated to control for multiple hypothesis testing. We included pairID as a cofactor in the model for the analyses with matched samples. We performed gene ontology (GO) enrichment analysis to identify GO terms associated with altered gene expression^24,25^. To perform pathway-level comparisons among lesional and non-lesional AD and controls, Gene Set Variation Analysis (GSVA) and GeneMANIA gene interaction network analysis were performed using previously published gene sets to analyze immune pathways^26^. Differentially expressed genes (DEGs) were defined as genes with a log2 fold change > 1 or < − 1, and FDR adjusted p-value < 0.05. In the population-level research, AD and control patients were 1:1 propensity score matched by age and sex. Systemic inflammatory markers were compared using the Student’s *t* test. AA and white AD patients were compared to their respective controls of matching race to control for inherent racial differences in systemic inflammatory markers.

## Results

A total of 18 skin biopsies were performed, including six lesional, six non-lesional, and six healthy controls (Supplemental Table 1). The average AD participant age was 51.8 (SD: 15.0) years, and 83.3% of participants were female. All participants identified as AA. Half of the AD participants self-reported severe itch, while the other half reported moderate itch as per the VRS. All controls reported no itch.

**Figure 1:**
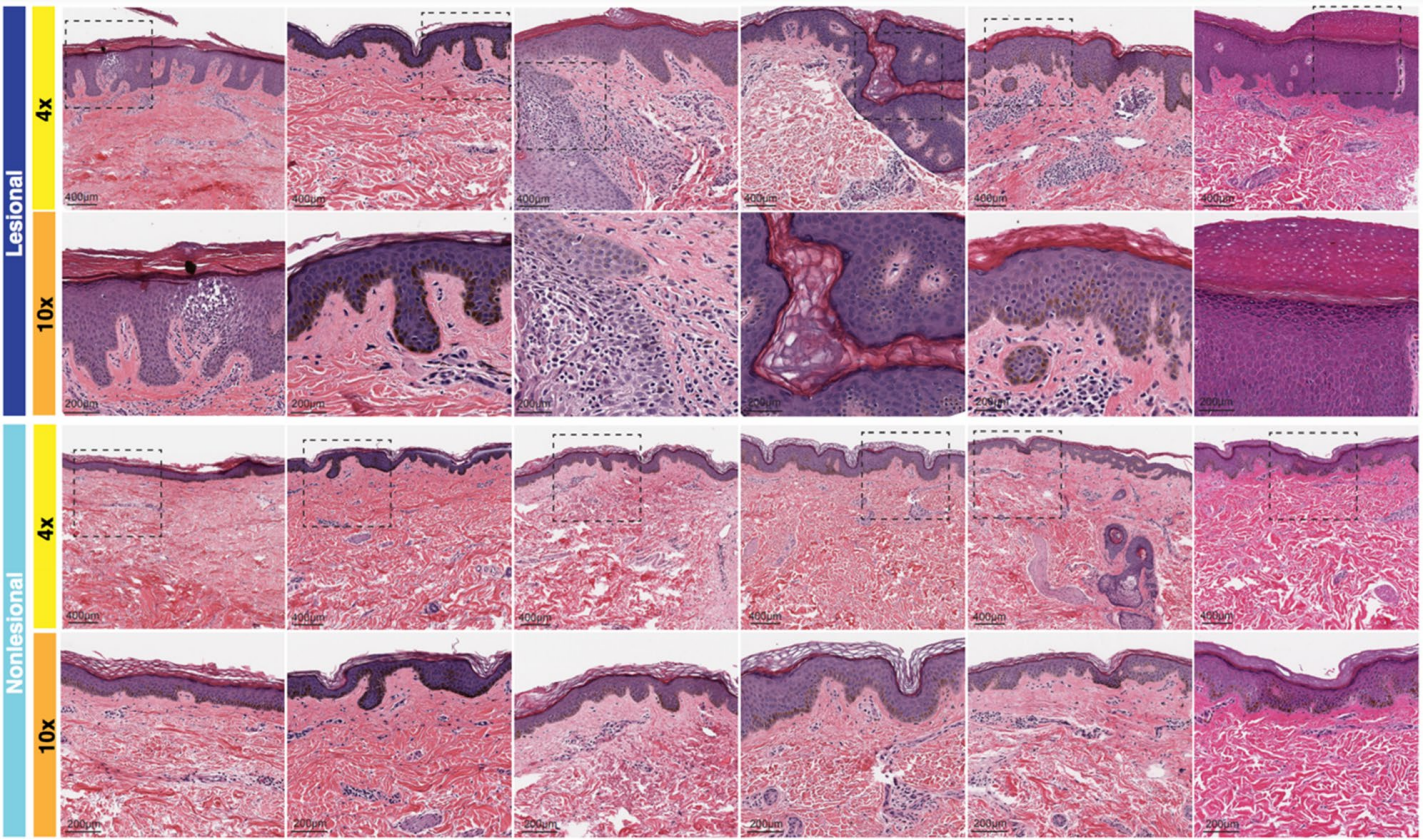
H&E of AD lesional and non-lesional skin. Magnification at × 4 and × 10 demonstrating psoriasiform and spongiotic dermatitis in lesional skin.

H&E stained tissue samples for lesional and non-lesional AD skin are shown in Fig. 1; patterns of psoriasiform and spongiotic dermatitis are observed in lesional AD skin. The epidermis of AD lesional skin averaged 101.67 (SD: 37.16) micrometers in thickness, significantly greater than the control skin epidermis, which averaged 46.67 (SD: 7.45) micrometers (p = 0.03) (Supplemental Figure 1A). Lesional skin received an average general inflammation grade of 1.67 (SD: 0.47), significantly greater than the average grade of 0.83 (SD: 0.37) of non-lesional skin (p = 0.04) and 0.17 (SD: 0.37) of control skin (p = 0.001). The average non-lesional skin grade was also significantly greater than the control skin grade (p = 0.03) (Supplemental Figure 1B).

**Figure 2:**
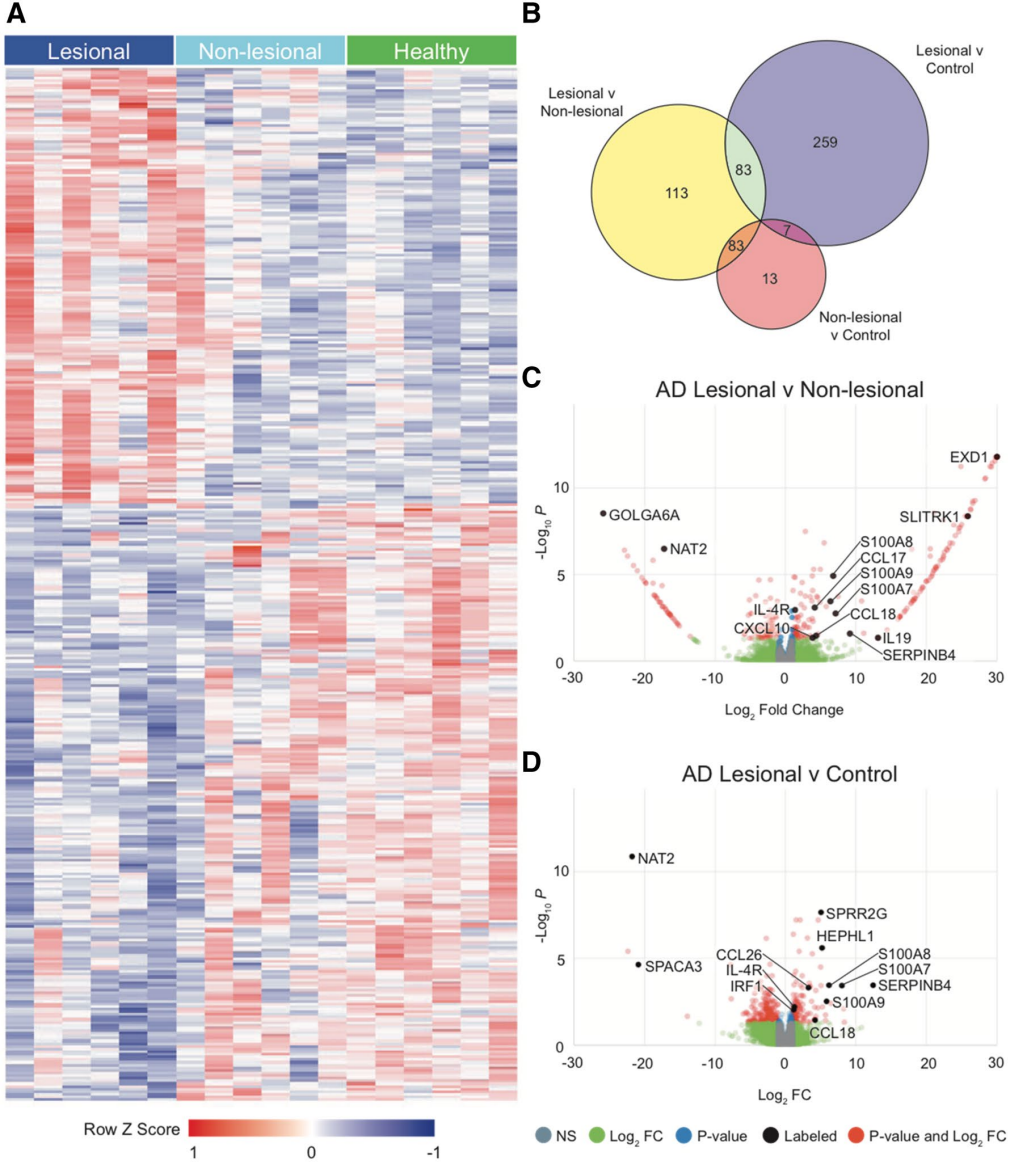
(**A**) Heatmap of gene expression by RNA-seq for differentially expressed genes (DEGs) between AD lesional vs. matched healthy control samples, where red is higher expression and blue is lower expression; DEGs defined as genes with a log base two fold change value less than -1 or greater than 1 and FDR adjusted p-value less than 0.05. (**B**) Venn diagram of DEGs for AD lesional samples, AD non-lesional samples, and matched healthy control samples. (**C**) AD lesional vs. non-lesional volcano plot. (**D**) AD lesional vs. control volcano plot. NS: not significant, FC: fold change, Log2FC: significant by Log2FC greater than 1 or less than − 1, *p-value*: significant by adjusted p-value < 0.05; *p-value and Log2FC*: significant by both adjusted p-value < 0.05 and Log2FC greater than 1 or less than − 1; heatmap was created with R version 4.0.2 (R Statistical Software) using the pheatmap package (https://cran.r-project.org/web/packages/pheatmap/index.html).

Sequencing of mRNA revealed variable expression of several genes among AD lesional, AD non-lesional, and healthy control skin samples. A visualization of the mRNA expression profile of the DEGs between AD lesional and healthy control skin is shown in Fig. 2A. There were 279 DEGs between lesional and non-lesional skin (L/NL), 349 between lesional and control skin (L/C), and 103 between non-lesional and control skin (NL/C). Of the DEGs identified, there were 83 in common between L/NL and L/C, 83 between L/NL and NL/C, and 7 between L/C and NL/C (Fig. 2B). Between lesional and non-lesional skin, the Th1, Th2, Th17, Th22 pathway-related genes that were differentially expressed included S100A7, S100A8, S100A9, SERPINB4, CCL17, CCL18, CXCL10, and IL4R (Fig. 2C). Between lesional and control skin, the Th1, Th2, Th17, Th22 pathway-related that were differentially expressed included S100A7, S100A8, S100A9, SERPINB4, CCL26, IL4R, IRF1, and CCL18 (Fig. 2D). In contrast, between non-lesional and control skin, there were no Th1, Th2, Th17, Th22 pathway-related genes that were differentially expressed.

**Figure 3:**
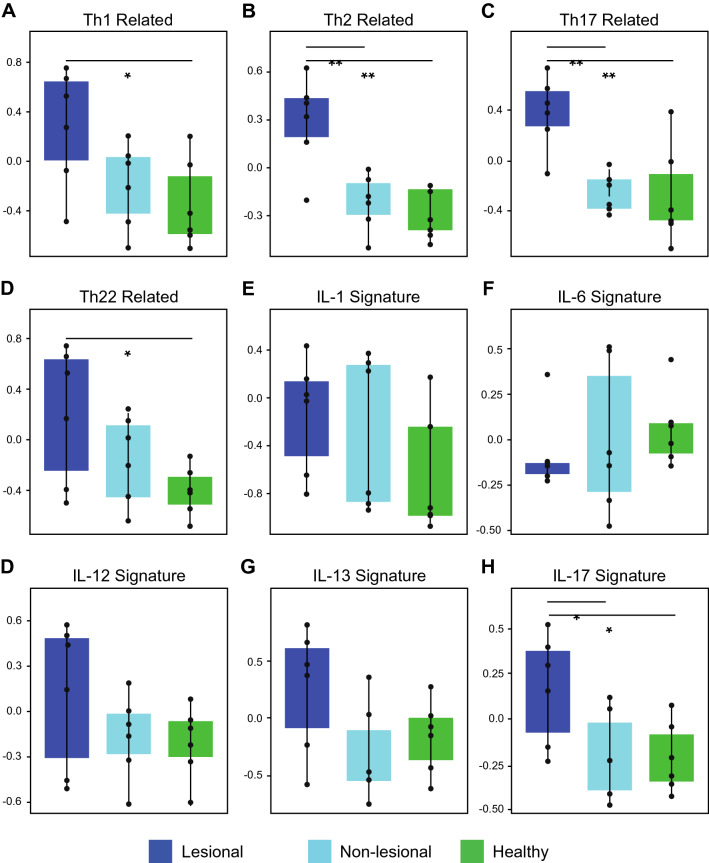
Gene set variation analyses (GSVA) for major immune pathways. *p < 0.05 and **p < 0.01 (n = 6 for each condition). Th1, Th2, Th17, Th22, and IL-17 signatures were upregulated in lesional skin.

**Figure 4:**
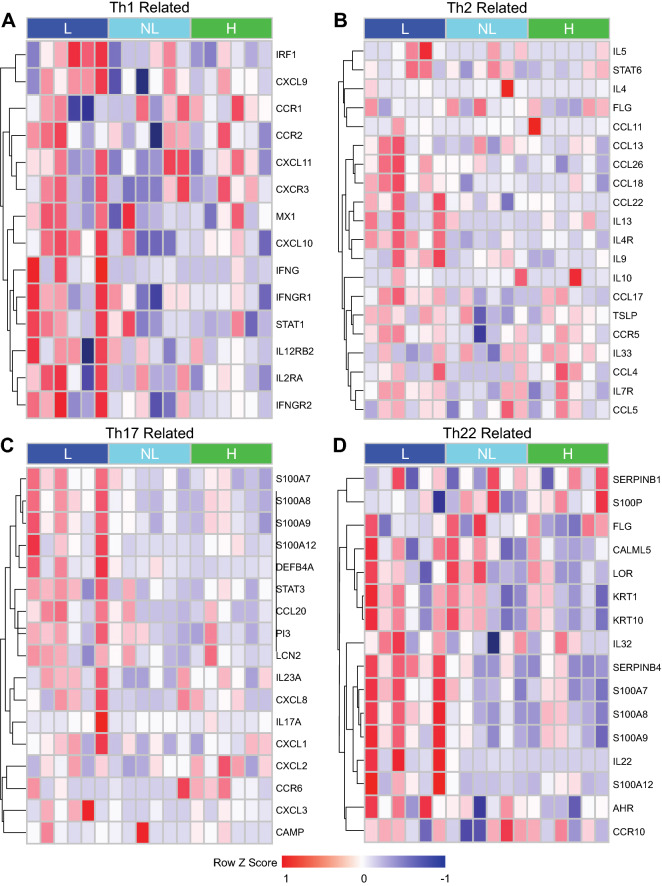
Heatmap of Th1-, Th2-, Th17-, Th22- related genes by mRNA-seq in AD lesional (L), AD non-lesional (NL), and matched healthy control (H) skin samples. Upregulation and downregulation denoted by red and blue, respectively. Heatmaps were created with R version 4.0.2 (R Statistical Software) using the pheatmap package (https://cran.r-project.org/web/packages/pheatmap/index.html).

**Figure 5:**
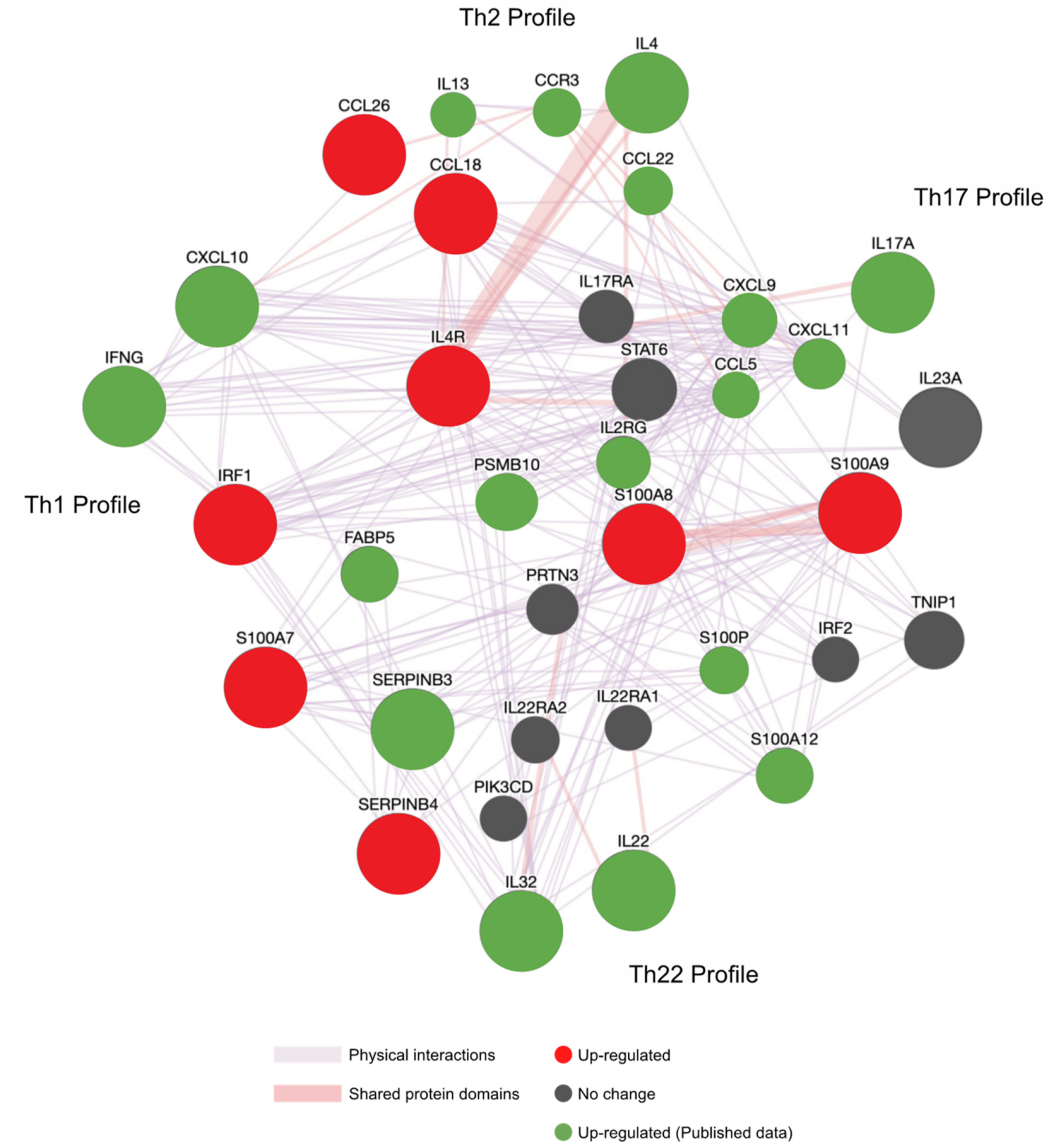
GeneMANIA functional association gene network for Th1-, Th2-, Th17-, Th22- related genes. For connecting lines, purple denotes physical interactions and red denotes shared protein domains.

GSVA for major immune pathways showed several patterns of immune polarization (Fig. 3). There was significant Th2, Th17, IL-17 upregulation in lesional skin compared to non-lesional (p = 0.006, p = 0.002, p = 0.04, respectively) and healthy skin (p = 0.003, p = 0.009, p = 0.03, respectively). Furthermore, Th1 and Th22 upregulation was observed in lesional versus healthy controls (p = 0.03 and p = 0.04, respectively) (Fig. 3). Differences in IL-1, IL-6, IL-12, and IL-13 related pathways did not reach statistical significance (Fig. 3). The GeneMANIA network provides a visualization of the shared protein domains and physical interactions of genes in the Th1, Th2, Th17, and Th22 pathways (Figs. 4, 5).

Gene ontology (GO terms) analysis of DEGs revealed enrichment of a diverse set of GO terms. The most enriched terms for upregulated DEGs of lesional versus non-lesional skin included adaptive immune response based on somatic recombination of immune receptors built from immunoglobulin superfamily domains (FDR 1.16 × 10^–16^), B cell-mediated immunity (FDR 3.02 × 10^–15^), and lymphocyte-mediated immunity (FDR 3.39 × 10^–15^). The most enriched terms for upregulated DEGs for lesional as compared to control skin included keratinization (FDR 3.07 × 10^–7^), keratinocyte differentiation (FDR 4.52 × 10^–7^), and cornification (FDR 5.51 × 10^–7^). The most enriched terms for downregulated DEGs of lesional versus control skin included a multicellular organismal process (FDR 3.97 × 10^–4^), anatomical structure development (FDR 4.74 × 10^–3^), and developmental process (FDR 9.03 × 10^–3^). The most enriched terms for downregulated DEGs of non-lesional versus control skin included B cell-mediated immunity (FDR 1.18 × 10^–17^), immunoglobulin mediated immune response (FDR 1.20 × 10^–17^), and humoral immune response mediated by circulating immunoglobulin (FDR 1.36 × 10^–17^) (Supplemental Tables 2–5).

In the population-level of analysis, a subset of AA and white AD patients had laboratory values for ESR (AA, n = 832; white, n = 2048), CRP (AA, n = 592; white, n = 1398), ferritin (AA, n = 737; white, n = 1312), and eosinophils (AA, n = 4573; white, n = 9984). The mean age of AD and control patients was 29.9 years (SD: 21.1) and 66% were female.

**Figure 6:**
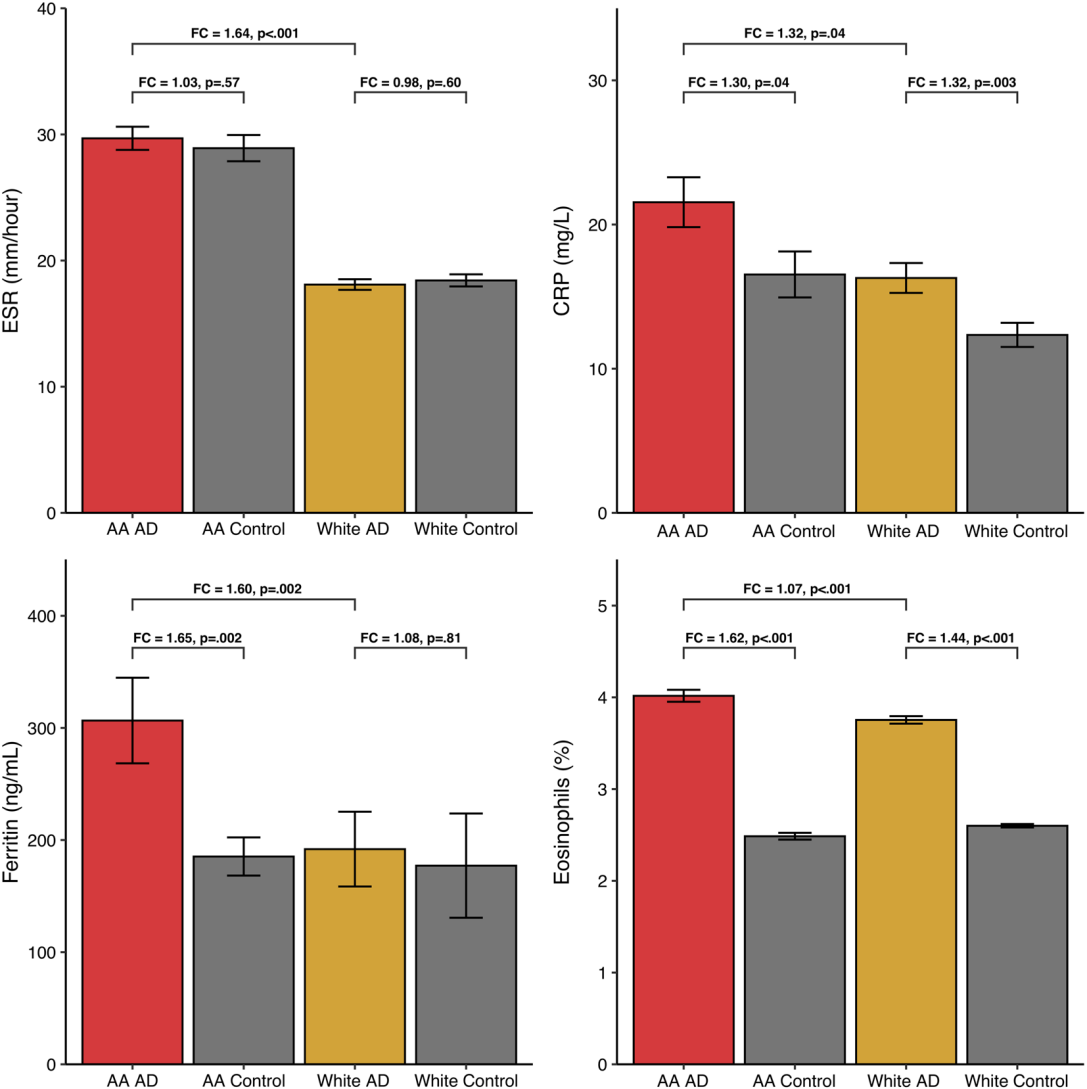
Population-level analysis from TriNetX: mean values for systemic inflammatory markers in AA and white AD and control patients. Error bars represent standard error of the mean. *FC* fold change.

As shown in Fig. 6, compared to age-, sex-, and race-matched controls, AA AD patients had higher values of CRP (FC 1.30, p = 0.04), ferritin (FC 1.65, p = 0.002), and blood eosinophils (FC 1.62, p < 0.001). Compared to white control patients, white AD patients had higher CRP (FC 1.32, p = 0.003) and blood eosinophils (FC 1.44, p < 0.001). Compared to white AD patients, AA AD patients had higher values of ESR (FC 1.64, p < 0.001), CRP (FC 1.32, p = 0.04), ferritin (FC 1.60, p = 0.002), and blood eosinophils (FC 1.07, p < 0.001).

## Discussion

Atopic dermatitis is traditionally associated with predominant Th2 polarization, with certain disease subtypes thought to be partially attributed to differential modulation of other immune pathways^27^. Our results corroborate existing evidence of Th2/Th22 skewing in AA AD while also demonstrating novel Th1 and Th17 cutaneous signatures that have not been previously described in this patient population.

Our findings suggest that the molecular signatures of AA AD patients reflect extensive immune activation. Czarnowicki et al*.* suggested a predominantly Th22-driven response in the epidermal hyperplasia and hyperkeratosis seen in AA AD patients^2^. Prurigo nodularis, which is associated with significant epidermal thickening and can be a manifestation of AD in subsets of AA patients, also was recently reported to have cutaneous Th22/Th17 polarization^28^. Similarly, our results demonstrate increased Th2 and Th22 induction in AA AD patients when samples were matched by age, race, gender, and biopsy-site location, providing an important robust confirmatory finding of a study showing Th2/Th22 bias in AA AD^29^. Additionally, murine models of human atopic dermatitis also suggest more broad immune involvement, including robust Th1, Th2, and Th17 activation^30^. Our findings also highlight the significant Th17 induction in AA AD lesions in addition to Th22 upregulation, suggesting the potential role that cutaneous Th17 induction may play in the epidermal hyperplasia seen in AA AD. GSVA of gene expression data demonstrates significant Th17 immune pathway enrichment in lesions compared to non-lesional samples and controls. Additionally, the S100 proteins, S100A7, S100A8, and S100A9, which have a role in epidermal hyperproliferation and are related to both Th17 and Th22 pathways^2,29,31^, were identified as DEGs in between lesional versus control and lesional versus non-lesional skin in our study. Additionally, alteration of immune function (i.e., B cell-mediated immunity, immunoglobulin mediated immune response, humoral immune response mediated by circulating immunoglobulin, and complement activation) observed through GO analysis comparing non-lesional vs. control DEGs may indicate global derangements in the skin of AA AD patients and predispose these individuals to a more lichenified phenotype^32^. With integrated gene network analysis, several chemokines and proteins were also shown as connections between the Th1, Th2, Th17, and Th22 pathways, and these intermediary molecules may facilitate the broad immune activation observed. The gene expression findings were corroborated by tissue sample histology which demonstrated features of Th17-driven psoriasiform dermatitis in combination with the classical Th2-driven spongiosis seen in AD^33–35^. Thus, our findings highlight the immune heterogeneity of AD in AA patients, and suggest that increased Th1 and Th17 upregulation in AA AD lesions, which has not been previously documented. The cutaneous findings observed in this study complement a report by Lang et al*.*, in which sub-Saharan AD patients were found to have higher serum levels of Th1 and Th17 cytokines/chemokines than European AD patients^36^. The cutaneous findings suggest broad systemic inflammation, which is corroborated by the finding of higher ESR, CRP, ferritin, and blood eosinophils in AA AD patients compared to white AD patients. As ESR, CRP, and ferritin are non-specific inflammatory markers for general inflammation, AA AD patients may have greater systemic inflammation encompassing multiple immune axes.

This immune signature may play a contributing role in the unique clinical morphology of AD observed in AA patients that have previously been overlooked. Current evidence suggests that the Th17 response is tied to a lichenified, skin thickened phenotype commonly found in psoriasis patients, but also more prominent in AA AD patients. As demonstrated by a study comparing lesions of psoriasis and AD, psoriatic lesions showed greater epidermal hyperproliferation and abnormal keratinocyte activation^37^. Among the Th17-related cytokines, IL-17 triggers abnormal keratinocyte proliferation and parakeratosis^38,39^, possibly explaining the connection between Th17 polarization and thickened AD lesions. Additional studies have further drawn striking similarities between psoriasis and the AD subtypes harboring Th17 bias. Pediatric AD patients are observed to have elevated Th17 upregulation and phenotypic changes characteristic of psoriasis lesions, including extensor and truncal distribution, increased keratinocyte activation with concomitant overexpression of keratin-related genes, and epidermal hyperplasia measured in both lesional and non-lesional skin^3^. Similar clinical findings have been observed in ethnic AD phenotypes with Th17 bias, such as Asian AD patients demonstrating increased lesional hyperplasia, parakeratosis, skin thickness, and truncal involvement^5,15,40^. Finally, with the enrichment of GO terms for keratinization and cornification along with the upregulation of SERPINB4 and S100 protein family genes^41–43^, this research highlights the potential role of increased barrier dysfunction contributing to the pathogenesis of AA AD.

Elucidation of AD immune pathways is an essential step for understanding disease pathogenesis and subsequent development and tailoring of therapies^44^. Immunomodulating biologic compounds have gained considerable traction for use in the treatment of AD, and recent studies have highlighted the janus kinase (JAK)-signal transducer and activation of transcription (STAT) signaling pathway as a target for drug development^14^. JAK-STAT has been implicated in the pathogenesis of AD by its induction of Th2 differentiation^45^. Trials have shown that new selective JAK inhibitors also suppress key itch-related cytokine signaling pathways needed for the Th1, Th2, and Th22 responses (including IL-4, IL-13, TSLP, IL-31, and IL-22) and are likely suitable treatment options for AD with broader immune activation^46–48^. Additionally, our findings of Th17 and IL-17 upregulation suggest that treatment options such as IL-17 inhibitors and Th17-targeting therapeutics may be beneficial and warrant further investigation. Therefore, the broad immune upregulation seen in AA AD patients in our study suggests that these patients may derive benefit from systemic immune therapies that modulate key drivers of multiple immune pathways.

The limitations of this study include low sample size and potential selection bias, as participants were chosen from patients with moderate to severe AD presenting to a tertiary care center. Because this study only included AA patients, meaningful comparisons of AA and non-AA AD cannot be made; however, the objective of the present study was to specifically study AA AD in depth as this group has been significantly understudied in the literature to date with limited published studies. Future studies and meta-analyses may pool patients from different ethnicities and races to obtain power for meaningful comparisons. In addition, advances in single-cell RNA-seq technology have allowed for higher resolution transcriptomic profiling, and future studies will rely on these analyses for insights as to the cell sources of the observed increased immune activation^49,50^. Additional research is also necessary to understand the impact of AD duration and therapeutics on the immune profile of AD patients, as these factors were not examined in this study.

In conclusion, our findings show that AD in AA patients may be associated with broader immune activation than previously thought. Despite prior studies demonstrating attenuation of the Th17-axis in AA AD, our results showed Th17-axis upregulation in addition to Th2 and Th22 induction. Th17 polarization in AD has been demonstrated in pediatric and Asian AD patients and is associated with a phenotypic subtype characterized by increased epidermal hyperplasia, lesion thickness, keratinocyte proliferation, and hyperkeratosis. These phenotypic features have been observed clinically in AA AD, further supporting the role of Th17 activation in the pathogenesis. These findings confirm the molecular heterogeneity of AD in AA patients and suggest that these patients may be responsive to systemic immune therapies that modulate multiple immune pathways. More recent understanding of T helper cell differentiation and plasticity also suggests the importance of further investigation of the potential for designing therapeutics that target patient-specific immune pathways driving the diverse manifestations of AD. Overall, more tailored approaches targeting the individual patient’s specific AD endotype are necessary to optimize AD therapeutics and improve patient outcomes. This study provides further insight into the immune dysregulation driving AD disease pathogenesis in AA patients and serves as a significant advancement in our understanding of potential areas of investigation for precision medicine in AD management.

## Supplementary Information

Supplementary Information.

## Data Availability

The datasets generated and analyzed during the current study are available from the corresponding author on reasonable request.
