# Supplementary material for: Transcriptomic analysis of atopic dermatitis in African Americans is characterized by Th2/Th17-centered cutaneous immune activation
Source: Sci Rep. 2021 May 27;11:11175. doi: 10.1038/s41598-021-90105-w (PMC8160001; doi:10.1038/s41598-021-90105-w)
Supplement: Supplementary file 1 — Supplementary Information. [file 41598_2021_90105_MOESM1_ESM.pdf]

Transcriptomic analysis of atopic dermatitis in African Americans is characterized by Th2/Th17-centered cutaneous immune activation

Supplementary Materials

Shannon Wongvibulsin, PhD[1,2], Nishadh Sutaria, BS[1], Suraj Kannan, BS[1,2], Martin Prince Alphonse, PhD[1], Micah Belzberg, MD[1], Kyle A. Williams, BS[1], Isabelle D. Brown, BS[1], Justin Choi, BA[1], Youkyung Sophie Roh, BA[1], Thomas Pritchard, MPH[1], Raveena Khanna, BA[1], Amarachi C. Eseonu, MD[1], Jaroslaw Jedrych, MD[1], Carly Dillen, PhD[1], Madan M. Kwatra, PhD[3], Anna L. Chien, MD[1], Nathan Archer, PhD[1], Luis A. Garza, MD, PhD[1], Xinzhong Dong, PhD[1,4], Sewon Kang, MD[1], Shawn G. Kwatra, MD[1*]

[1]Johns Hopkins University School of Medicine, Department of Dermatology, Baltimore, MD
[2]Johns Hopkins University School of Medicine, Department of Biomedical Engineering, Baltimore, MD
[3]Department of Anesthesiology, Duke University School of Medicine, Durham, NC, USA
[4]The Solomon H. Snyder Department of Neuroscience, Center for Sensory Biology, Johns Hopkins University School of Medicine, Baltimore, MD

*Corresponding author.
Shawn G. Kwatra
Email: skwatra1@jhmi.edu
Tel: 410-955-8662
Cancer Research Building II, Johns Hopkins University School of Medicine, Suite 206
1550 Orleans Street
Baltimore, MD 21231, USA

Supplemental Table 1: Participant Characteristics

Characteristic	Atopic Dermatitis Patients (n=6)	Control Patients (n=6)
**Age (years), mean (SD)**	51.8 (15.0)	51.5 (13.1)
**Sex, Number (%)**		
Female	5 (83.3)	5 (83.3)
Male	1 (16.7)	1 (16.7)
**Race/Ethnicity, Number (%)**		
African American	6 (100)	6 (100)
**Itch Severity, Number (%)**		
Severe	3 (50)	0 (0)
Moderate	3 (50)	0 (0)

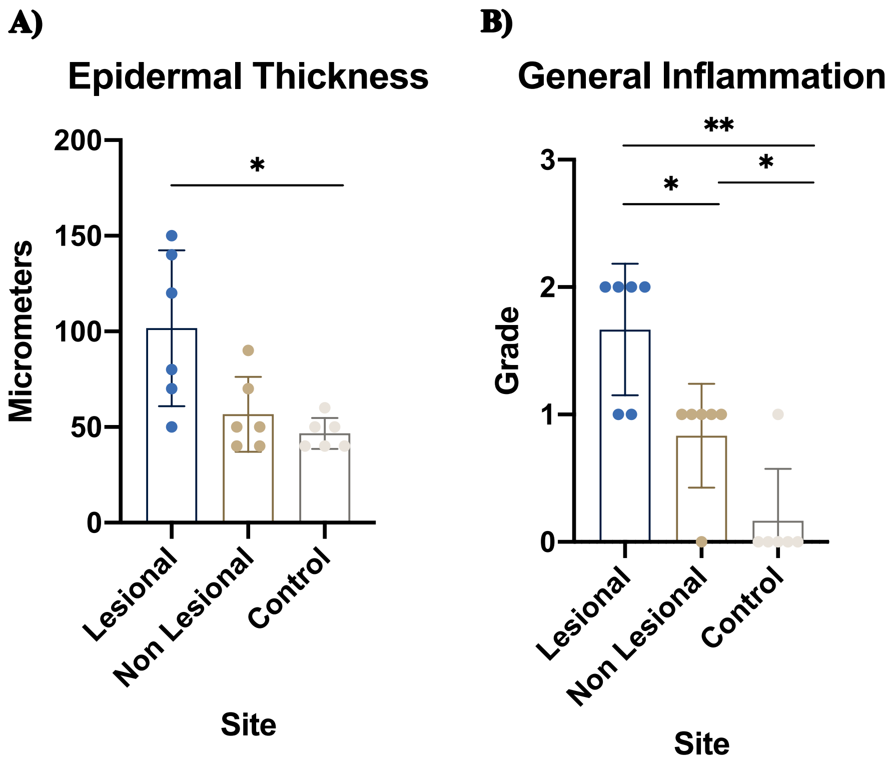

Supplemental Figure 1: A) Epidermal thickness and B) general inflammation for AD lesional, non-lesional, and control skin. * denotes $p < 0.05$ and ** denotes $p < 0.01$

Supplemental Table 2: Top 5 GO Terms for Lesional vs. Non-Lesional Upregulated DEGs

GO Term	FDR
adaptive immune response based on somatic recombination of immune receptors built from immunoglobulin superfamily domains (GO:0002460)	1.16E-16
B cell-mediated immunity (GO:0019724)	3.02E-15
lymphocyte-mediated immunity (GO:0002449)	3.39E-15
immunoglobulin mediated immune response (GO:0016064)	4.50E-15
defense response to other organism (GO:0098542)	4.96E-14

GO Terms for Lesional vs. Non-Lesional Downregulated DEGs

No statistically significant results

Supplemental Table 3: **Top 5 GO Terms for Lesional vs. Control Upregulated DEGs**

GO Term	FDR
keratinization (GO:0031424)	3.07E-07
keratinocyte differentiation (GO:0030216)	4.52E-07
cornification (GO:0070268)	5.51E-07
epidermal cell differentiation (GO:0009913)	2.35E-06
defense response (GO:0006952)	2.67E-06

Supplemental Table 4: **Top 5 GO Terms for Lesional vs. Control Downregulated DEGs**

GO Term	FDR
multicellular organismal process (GO:0032501)	3.97E-04
anatomical structure development (GO:0048856)	4.74E-03
developmental process (GO:0032502)	9.03E-03
system process (GO:0003008)	1.17E-02
multicellular organism development (GO:0007275)	1.37E-02

GO Terms for Non-lesional vs. Control Upregulated DEGs

No statistically significant results

Supplemental Table 5: **Top 5 GO Terms for Non-Lesional vs. Control Downregulated DEGs**

GO Term	FDR
B cell-mediated immunity (GO:0019724)	1.18E-17
immunoglobulin mediated immune response (GO:0016064)	1.20E-17
humoral immune response mediated by circulating immunoglobulin (GO:0002455)	1.36E-17
complement activation, classical pathway (GO:0006958)	1.44E-17
complement activation (GO:0006956)	1.50E-17
